# 

**Published:** 1885-08

**Authors:** 


### CONTENTS:

Art. I—Am. Med. Association, Section of Dental and Oral Surgery................145

Art. II—The Teeth of Different People, By Parsons Shaw, D. D. S...............164

Art. III—Chemistry in Life and in Death, By George Watt, M. D., D. D. S........171

Art. IV—The Reckless Sacrifice of Tooth Substance in Devitalized Teeth, By W. N. Morrison, D. D. S........180

Art. V—A Common Sense Pivot Tooth, By Dr. J. C. Adams........183

Art. VI—The Poisonous Effects of Amalgam Fillings, By Thomas Fletcher, F. C. S.185

### EDITORIAL, ETC.

The Practice of Dentistry in Germany. Translated for Am. Journal of Dental Science, By Emil Amend, Student in Dental Department, University of Maryland........187

### MONTHLY SUMMARY.

Galvanism for Neuralgia........191

Effect on Offspring of Consanguineous Marriages........192

